# *Sarcocystis* spp. of New and Old World Camelids: Ancient Origin, Present Challenges

**DOI:** 10.3390/pathogens13030196

**Published:** 2024-02-23

**Authors:** Sarah N. Wieser, Susana M. Giuliano, Juan Reategui Ordoñez, Ximena Barriga Marcapura, Luis V. M. Olivera, Miguel Angel Chavez Fumagalli, Leonhard Schnittger, Mónica Florin-Christensen

**Affiliations:** 1 Instituto de Patobiología Veterinaria, CICVyA, Instituto Nacional de Tecnología Agropecuaria (INTA), Hurlingham B1686, Argentina; wieser.sarah@inta.gob.ar (S.N.W.); schnittger.leonhard@inta.gob.ar (L.S.); 2 Consejo Nacional de Investigaciones Científicas y Técnicas (CONICET), Buenos Aires C1425FQB, Argentina; 3 Facultad de Ciencias Veterinarias, Universidad de Buenos Aires, Buenos Aires C1427CWN, Argentina; smgiulia@gmail.com; 4 Laboratorio de Biotecnología Animal, Vicerrectorado de Investigación, Universidad Católica de Santa María, Arequipa 04000, Peru; jreategui@ucsm.edu.pe (J.R.O.); xbarriga@ucsm.edu.pe (X.B.M.); 5 Facultad de Medicina Veterinaria, Universidad Nacional del Altiplano, Puno 21001, Peru; lvolivera@unap.edu.pe; 6 Computational Biology and Chemistry Research Group, Vicerrectorado de Investigación, Universidad Católica de Santa María, Arequipa 04000, Peru

**Keywords:** South American camelids, Old World camels, *Sarcocystis*, sarcocysts

## Abstract

*Sarcocystis* spp. are coccidian protozoans belonging to the Apicomplexa phylum. As with other members of this phylum, they are obligate intracellular parasites with complex cellular machinery for the invasion of host cells. *Sarcocystis* spp. display dixenous life cycles, involving a predator and a prey as definitive and intermediate hosts, respectively. Specifically, these parasites develop sarcocysts in the tissues of their intermediate hosts, ranging in size from microscopic to visible to the naked eye, depending on the species. When definitive hosts consume sarcocysts, infective forms are produced in the digestive system and discharged into the environment via feces. Consumption of oocyst-contaminated water and pasture by the intermediate host completes the parasitic cycle. More than 200 *Sarcocystis* spp. have been described to infect wildlife, domestic animals, and humans, some of which are of economic or public health importance. Interestingly, Old World camelids (dromedary, domestic Bactrian camel, and wild Bactrian camel) and New World or South American camelids (llama, alpaca, guanaco, and vicuña) can each be infected by two different *Sarcocystis* spp: Old World camelids by *S. cameli* (producing micro- and macroscopic cysts) and *S. ippeni* (microscopic cysts); and South American camelids by *S. aucheniae* (macroscopic cysts) and *S. masoni* (microscopic cysts). Large numbers of Old and New World camelids are bred for meat production, but the finding of macroscopic sarcocysts in carcasses significantly hampers meat commercialization. This review tries to compile the information that is currently accessible regarding the biology, epidemiology, phylogeny, and diagnosis of *Sarcocystis* spp. that infect Old and New World camelids. In addition, knowledge gaps will be identified to encourage research that will lead to the control of these parasites.

## 1. Introduction

Sarcocystosis is a parasitic infection caused by different species of protozoans belonging to the *Sarcocystis* genus. With over 200 described species, different *Sarcocystis* spp. infect mammals, including humans, as well as birds and reptiles. Their life cycles involve a predator and a prey that serve as definitive and intermediate hosts, respectively. Typical of the intermediate host phase of *Sarcocystis* is the formation of sarcocysts—wall-surrounded capsules in which the parasites divide asexually—which may be micro or macroscopic, depending on the species [1].

**Figure 1:**
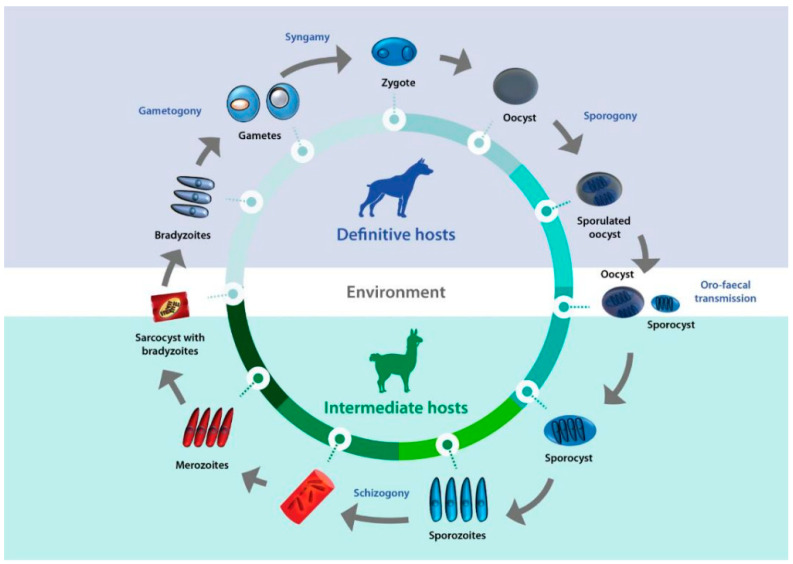
Life cycle of *Sarcocystis*. A typical life cycle of *Sarcocystis* species is shown, exemplified with a llama and a dog as intermediate and definitive hosts, respectively.

*Sarcocystis* complex life cycles alternate between invading, dividing, and sexual stages. After a predator ingests the meat of a prey containing sarcocysts, bradyzoites—the infective stage borne in these structures—invade the goblet cells of the predator’s intestine. Micro- and macrogametes are formed, and gamete fusion leads to the formation of an oocyst which, after being excreted with the feces into the environment, sporulates to form four sporozoites. Intermediate hosts ingest oocysts when grazing or drinking contaminated pasture or water, and enzymes in their intestine lead to the liberation of sporozoites. Individual sporozoites can also be found in stools due to occasional breakage of the thin oocyst wall, and can contaminate water and pastures, which leads to direct ingestion of sporozoites by the intermediate host. Once in the intestinal lumen, sporozoites invade the endothelial cells of mesenteric lymph node arteries, where they reproduce asexually by schizogony, forming schizonts with lobed nuclei that have the appearance of a rosette. Merozoites eventually bud off and are released into the bloodstream, disseminating the infection in the intermediate host. They can be found free or inside mononuclear cells, where they undergo binary division by endodyogeny. When merozoites invade the endothelial cells of downstream arterioles, capillaries, and veins, a new cycle of schizogony is initiated. In turn, invasion of a myocyte or a nervous cell by a merozoite leads to the formation of sarcocysts, the final stage in the intermediate host. The parasite remains inside a parasitophorous vacuole (PV), and the parasitophorous vacuolar membrane (PMV) together with parasite secretions form a wall that provides a safe microenvironment for parasite multiplication. Depending on the *Sarcocystis* species, final-destination cells can be myocytes of skeletal or cardiac muscles, or neural cells. In the sarcocysts, merozoites transform into metrocytes, which rapidly divide by endodyogeny and eventually turn into infective bradyzoites [1,2,3] (Figure 1).

Most *Sarcocystis* spp. that infect livestock have a worldwide distribution and, in general, occur with high prevalence in both industrialized and developing countries [3]. The economic burden of *Sarcocystis* infections of livestock is related to abortions, low meat and/or milk yield, poor body growth, and outbreaks of clinical sarcocystosis that can be fatal. Additionally, carcasses destined for human consumption can be condemned by sanitary authorities when abundant macroscopic *Sarcocystis* spp. sarcocysts or *Sarcocystis* spp.-associated lesions due to eosinophilic myositis are encountered. Eosinophilic myositis is an inflammatory condition of striated muscles that leads to necrosis of the affected areas which has been described in cattle and some other mammals [1,4,5]. For most *Sarcocystis* species, especially those infecting wild animals, their effect on host fitness is unknown.

Camelids are Artiodactyla mammals grouped in the Camelidae family, the only extant family of the Tylopoda suborder (from the Greek: “feet with cushions”). They regurgitate and rechew food but are not ruminants and are differentiated from the latter by several anatomical features, including their stomach with three compartments, lack of horns, and the presence of real canine teeth and plantar cushions, as well as other physiological and behavioral features. Importantly, camelids differ from ruminants in their susceptibility to microbial and parasitic diseases [6].

Old World camelids (OWCs) belong to the genus *Camelus*, which comprises three extant species: *C. dromedarius* (one-humped camel or dromedary), *C. bactrianus* (two-humped or Bactrian camel), both of which have been domesticated, and the wild and highly endangered *C. ferus* (two humps). The population size of OWCs has been estimated to be at least 35.5 million heads, of which 95% are dromedaries [7]. South American camelids (SACs) comprise four species: the wild *Lama guanicoe* (guanaco) and *Vicugna vicugna* (vicuña), and the domesticated *Lama glama* (llama) and *Vicugna pacos* (alpaca), with an overall estimated population of 10 million heads [8]. Notably, llamas and alpacas have been introduced to farms in some European countries, South Africa, and Australia and, thus, their geographic distribution and numbers of heads are considerably larger than those mentioned here [7,8].

Both OWCs and SACs are adapted to harsh environmental conditions, including extreme temperatures, intense solar radiation, water scarcity, and poor pastures. Under such rough settings, most other livestock species are either unable to thrive or show a significant decline in production. Camelids thus constitute an attractive livestock choice in a scenario of climate change, shortage of water, and reduction in agricultural areas [9].

Camelids have a long history of association with humans. Human groups who lived as gatherers and hunters must have found in camels a good source of food and hides, and through their domestication some 3 to 7 thousand years ago (kya), they became important suppliers of vital goods to ancient civilizations in the Old and New Worlds [10,11]. Currently, camelids continue to be an important asset to a considerable number of human populations, and among other benefits, their meat is a valuable source of animal protein and an attractive product for the gourmet cuisine [12,13,14].

Both OWCs and SACs act as intermediate hosts for some *Sarcocystis* spp., sometimes with a relevant negative impact on local economies [15,16,17,18]. This review will present the available information on *Sarcocystis* spp. that infect camelids in the Old and the New World, draw parallels between these two scenarios, and analyze the phylogenetic relationships among these parasites.

## 2. *Sarcocystis* Infecting OWCs and SACs

Four *Sarcocystis* spp. have so far been described to infect camelids: *S. cameli* and *S. ippeni* for OWCs, and *S. aucheniae* and *S. masoni* for SACs [3,17,19].

The first observation of *Sarcocystis* infections in OWCs was carried out in Egypt by Mason (1910), who reported macroscopic sarcocysts in the muscles of camels and used the name *S. cameli* to refer to the etiological agent. Later, several additional case reports of *Sarcocystis* species infecting OWCs appeared in the literature, which were designated as *S. ippeni*, *S. camelicanis*, *S. camelocanis*, and *S. miescheri*, depending on their sarcocyst wall or oocyst features. A taxonomic revision of OWCs sarcocystosis led to the acceptance of *S. cameli* and *S. ippeni* as valid species, while *S. camelicanis*, *S. camelocanis*, and *S. miescheri* were considered synonymous with *S. cameli*. Importantly, the vast majority of *Sarcocystis* reports are from dromedary camels [16,19,20].

In the case of SAC, the first description of a macroscopic sarcocyst in a llama took place in 1913, and the corresponding parasite was named *S. aucheniae* [21]. Later, the names *S. tilopodi* and *S. guanicoecanis* were used for parasites forming sarcocysts in guanaco [22,23]. A *Sarcocystis* species forming microscopic cysts in SACs was initially named *S. lamacanis* [24,25]. Electron microscopy and molecular studies established *S. aucheniae* as the only species forming macroscopic cysts in llamas, alpacas, and guanacos, while the species forming microscopic cysts was redescribed as *S. masoni* in honor of Dr Eugene Mason. Thus, the names *S. tilopodi*, *S. guanicoecanis*, and *S. lamacanis* are currently considered invalid [3,17,26].

In different *Sarcocystis* spp., sarcocysts vary in shape (globular, filamentous, fusiform), size, and other characteristics, such as the presence or absence of internal partitions and variations in their wall ultrastructure [2,3]. In the case of *Sarcocystis* spp. that infect camelids, both *S. aucheniae* and *S. cameli* generate macroscopic sarcocysts that are visible to the naked eye (oval, 5–20 mm × 2 mm, and fusiform, 1.5–5 mm × 0.2–0.4 mm, respectively). Additionally, microscopic cysts of *S. cameli* (700 × 100 µm) are commonly found in camel tissues. In turn, only microcysts were described for *S. masoni* (fusiform, 800 × 95 µm) and *S. ippeni* (globular, 100–120 × 50–100 µm) [3,17,19] (Table 1).

In all *Sarcocystis* spp., the sarcocyst wall essentially consists of the PVM covering a granular, electron-dense layer from which septa can arise. When present, septa cross the cyst, separating its cavity into compartments, where metrocytes and bradyzoites are found. The number of parasites contained in a sarcocyst varies with the species and the stage of maturation: young cysts as small as 5 μm in diameter might contain only two parasites, while a mature macroscopic cyst can contain 20 million, as has been observed for *S. aucheniae* [3,26].

The sarcocyst wall can remain relatively simple in some species and, in others, form projections (villar protrusions) of different sizes and shapes that bulge outwardly and can contain microfilaments, microtubules, electron-dense bodies, minute granules, and small vesicles [1,3]. At least 82 ultrastructural types of cyst wall have been described for different *Sarcocystis* spp. [3]. Notably, the cyst walls of both *S. cameli* and *S. masoni* have a common “9j” conformation, characterized by the presence of upright finger-like villar protrusions with knob-like structures arising from the PVM, in which microtubules can be observed [3,17]. *S. aucheniae* presents a ‘type 21’ sarcocyst wall ultrastructure, with highly branched cauliflower-like villar protrusions, similar to that of *S. gigantea* [1,3,17]. Finally, *S. ippeni* has a characteristic ‘type 32’ sarcocyst wall structure. This type of ultrastructure is characterized by thorn-like villar protrusions with microtubules radiating into the granular layer and has not been previously described in any other *Sarcocystis* species [3] (Table 1).

**Figure 2:**
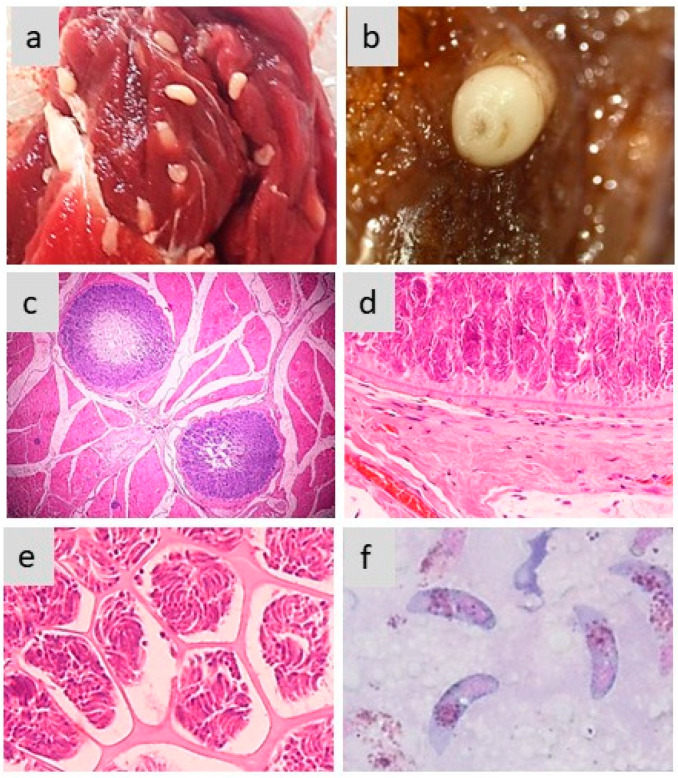
Morphology of sarcocysts and bradyzoites in *S. aucheniae*. (**a**,**b**) Macroscopic sarcocysts in llama (**a**) and alpaca (**b**) skeletal muscle; (**c**) hematoxylin eosin-dyed cross-section of alpaca skeletal muscle with two macroscopic sarcocysts, in which zoites are located to the periphery and the center is empty (100×); (**d**,**e**) details of hematoxylin eosin-dyed section of a macroscopic sarcocyst showing the morphology of the cell wall (**d**) and compartments with thousands of banana-shaped bradyzoites (**e**) (400×); (**f**) bradyzoites observed in a cyst stained with Giemsa (1000×). The photographs were obtained by S.N.W. and L.V.M.O.

Figure 2 exemplifies the different aspects of sarcocysts produced by *S. aucheniae* in the skeletal muscles of llamas and alpacas.

## 3. Definitive Hosts

Determination of the definitive host(s) of camelid *Sarcocystis* spp. has so far been based on experimental infection studies. As will be briefly related in the next paragraphs, these studies have indicated that the domestic dog (*Canis familiaris*) can act as a definitive host of *S. aucheniae*, *S. masoni*, and *S. cameli*. On the other hand, no reports on definitive hosts for *S. ippeni* are available. In most cases, microscopic observation of oocysts or sporocysts in feces has been used as conclusive evidence. However, confirmatory studies in which the species of the excreted parasites is determined by molecular methods would be desirable.

In the case of *S. aucheniae*, dogs, cats, rats, and mice were fed with raw guanaco meat containing macrocysts. Sporocysts were later only observed in dog feces [23]. Excretion of *Sarcocystis* sp. sporocysts in the feces of dogs fed with SAC meat was also confirmed by other authors [30,31,32].

For *S. masoni*, experimental infection has also determined that dogs can serve as a definitive host. In this case, the species of excreted oocysts was confirmed by PCR-RFLP analysis of the cytochrome c oxidase subunit I (*cox-1*) gene [33].

In turn, *S. cameli* sporocysts were observed in the feces of dogs fed with dromedary meat infested with sarcocysts of this species [34,35,36,37].

Histological studies of the intestines of dogs fed with *S. masoni*- or *S. aucheniae*-infected SAC meat have shown that oocysts and sporocysts were mostly concentrated towards the tips of the villosities, without further alterations in the mucosa [32,33]. Gamogony was observed within the lamina propria of the intestines of puppies that had been fed with *S. cameli*-infected meat [38]. *S. cameli* zygotes were first observed in the lamina propria 24 h after meat ingestion, and sporulated oocysts were evident in feces at 7–13 days post-ingestion [34,35,36,37,38,39]. Prepatent periods of 8–9 days and 9–16 days were observed for *S. masoni* and *S. aucheniae*, respectively [30,31,32].

The consumption of *S. aucheniae* sarcocysts has been associated with toxicity and gastroenteritis signs in dogs and rabbits [40,41]. In the case of dogs, these effects could be partly due to the colonization of the intestinal mucosa by parasites. However, it is also important to take into account that *Sarcocystis* spp. parasites produce a thermosensitive peptidic endotoxin, known as sarcocystin, which has been described for *S. aucheniae* as well as for *S. fayeri* that infects horses, *S. wapiti* and *S. sybillensis* that infect sika deer, and *S. cruzi* that infects cattle [1,40,41,42,43,44].

## 4. Pathogenesis of Sarcocystosis in Camelids

There is a paucity of information on the pathogenic effects of sarcocystosis in camelids. Infections generally appear to be asymptomatic, although a few clinical cases have been reported. In SAC, two cases of acute sarcocystosis (Dalmeny’s disease) in alpacas have been published. In one of them, a 6-year-old pregnant alpaca imported to the USA aborted and died shortly after exhibiting lethargic behavior, marked muscle tremors, and respiratory distress. Necropsy revealed numerous cysts in the skeletal muscles. Inflammatory disease of the muscles was demonstrated, mostly caused by leukocytes, especially eosinophils accumulation (eosinophilic myositis), and thought to be associated with old, degenerating *S. aucheniae* sarcocysts [45].

Another case of sarcocystosis-related myositis was described in an alpaca born and raised in a farm in Australia. The alpaca was presented with multiple subcutaneous abscesses. Histologic examination revealed necrotizing and histiocytic myositis and cellulitis, as well as central caseation and numerous microscopic sarcocysts. However, the species of the sarcocyst-forming parasite was not identified [46].

Experimental oral infections of alpacas and camels with high doses of *S. masoni* or *S. cameli* sporocysts, respectively, have led to acute sarcocystosis with anorexia, lethargy, and anemia. Fatal cases were registered, and hemorrhages in several organs were observed upon necropsy [47,48,49]. Importantly, these are extreme cases since the numbers of sporocysts utilized (≥250,000 per animal) are likely to largely surpass the amounts ingested in a natural infection with contaminated pastures or water. In a study performed in slaughtered dromedaries from Iran, different degrees of inflammatory responses were observed in tissues surrounding old, degenerating microscopic *Sarcocystis* sp. sarcocysts, with infiltration of macrophages, lymphocytes, plasma cells, eosinophils, and fibroblasts [50]. In agreement with an inflammatory response, expression of the interleukin-6 gene was significantly increased in the *Sarcocystis* sp. microcyst-infected tongue and diaphragm tissues of dromedaries from Saudi Arabia [51].

Overall, these investigations demonstrate that *Sarcocystis* spp. may cause considerable disease in SACs and OWCs, although infections are generally subclinical. More research is needed to understand the pathogenesis of both macro- and microscopic sarcocystosis, as well as their influence on musculoskeletal and cardiac function, immunity, well-being, and the productivity of infected camelids [15,16].

## 5. Diagnosis

No commercial or validated diagnostic tools for sarcocystosis applicable to live camelids are available so far, and diagnosis is currently carried out post-mortem. Macroscopic sarcocysts of *S. aucheniae* or *S. cameli* can be observed upon visual inspection of skeletal muscles, which is the current procedure used in abattoirs to establish if an animal is infected [52,53]. In the case of microscopic cysts, different detection methods can be applied, including muscle squash, pepsin or trypsin digestion, histopathological examination, and in some cases electron microscopic studies. However, these methods are only employed for research and not for routine examinations of camelid carcasses [17,38].

PCR amplification followed by sequencing of different molecular markers, such as the 18S and 28S ribosomal RNA (rRNA), *cox-1* genes, and the ITS region, as well as PCR-RFLP of *cox-1* or 18S rRNA genes, has been used for the identification and molecular characterization of SACs and OWCs *Sarcocystis* spp. [17,26,27,33]. These methods are not practical for diagnostic purposes and are meant to be applied to tissue or cyst samples obtained after necropsy. No nucleotide sequences, on the other hand, are so far available for *S. ippeni*; thus, molecular characterization of this parasite is still pending.

Recently, highly sensitive seminested PCR protocols for *S. aucheniae* were developed based on the parasite’s 18S rRNA gene. Using these techniques, it was possible to detect *S. aucheniae* DNA in the blood of live llamas from Argentina [54,55]. Primers were designed to specifically amplify *S. aucheniae* DNA, avoiding cross-amplification of the DNA of *Toxoplasma gondii* or *Neospora caninum*, two closely related coccidians that also infect SACs [56]. In addition, the recent availability of whole-length 18S rRNA sequences of *S. masoni* has allowed for confirmation that these PCR detection protocols of *S. aucheniae* do not cross-react with other SAC-infecting *Sarcocystis* species [33].

One of these PCRs has a duplex format to simultaneously amplify a segment of the host mitochondrial 16S rRNA gene, which serves as a positive control for successful DNA extraction and amplification. To evaluate the usefulness of this method for diagnosing *S. aucheniae* infection, it was applied to detect parasite DNA in the blood of 80 live llamas destined for meat consumption. The presence of macroscopic cysts was analyzed postmortem by visual inspection of the carcasses. The results showed no correlation between DNA and sarcocyst detection. The observed PCR-positive/sarcocyst-negative animals might correspond to early infections in which sarcocysts have not yet been formed, while PCR-negative/sarcocyst-positive animals might correspond to older infections in which parasites are confined to muscles and do not circulate in the blood [55]. In any case, *S. aucheniae* parasitemia appears to always be very low and, thus, the possibility of the continuous detection of parasite DNA in blood using more sensitive methods—when available—cannot be overruled.

Antibodies against *Sarcocystis* spp. antigens evidence the previous exposure of an animal to the parasite. This notion was used to develop a fixed indirect immunofluorescence test (IFAT) using *S. aucheniae* whole bradyzoites as antigens. The sera of most of the studied llamas (77%) from the province of Jujuy, Argentina, reacted with the parasites, indicating the high seroprevalence of SAC sarcocystosis in this area. The same sera also recognized bradyzoites of *S. cruzi*, a bovine-infecting *Sarcocystis* sp., with an even higher prevalence (92.5%), indicating cross-reactivity at the genus level which was likely due to *S. masoni* infections [56].

An indirect ELISA (iELISA) was also established using a 23 kDa immunogenic protein fraction of *S. aucheniae* sarcocysts as antigen [57]. This assay detected seroprevalence values in llamas in Argentina ranging from 23 to 50% depending on management conditions. This serological method is less labor-intensive than IFAT and allows for the processing of a large number of samples in a time-efficient manner. However, because there is no established gold standard for the serological detection of *S. aucheniae*, the sensitivity and specificity of this iELISA cannot yet be determined. In addition, the correlation between serologic detection and the presence of sarcocysts is yet to be investigated.

An important constraint of both IFAT and iELISA is the use of parasites or sarcocyst protein fractions as antigens, which might bring restrictions in reproducibility and material accessibility. Thus, the identification of immunodominant conserved antigens suitable for the development of serological tests based on recombinant or chemically synthesized peptides is highly desirable. The recent sequencing of the *S. aucheniae* sarcocyst transcriptome has provided a pool of attractive targets for the development of diagnostic tools. Indeed, in silico analysis of the transcriptome unraveled an array of proteins predicted to be anchored to the cell membrane through glycosylphosphatidylinositol (GPI) anchors. This type of protein was shown to be generally species-specific and immunodominant in other pathogenic protozoa, two requirements for serological tests [58]. These features, as well as their conservation among parasite geographical isolates, needs experimental confirmation. In addition, an immunoproteomic approach was recently carried out for *S. aucheniae*, in which soluble immunoreactive proteins present in sarcocysts were sequenced by mass spectrometry and identified by in silico searches in the transcriptome. Highly antigenic B-cell epitopes were predicted in silico, and those that showed good water solubility and a low probability of cross-reactivity with other coccidia were shortlisted for future development in peptide-based serological methods [59].

An important objective of ongoing research is the development of low-cost and reproducible diagnostic methods that can predict the presence of cysts in live camelids as this will result in a significant advance in the control of SAC sarcocystosis.

## 6. Epidemiology and Risk Factors

Macroscopic sarcocysts of *S. aucheniae* have been detected, mostly in intercostal and cervical skeletal muscles, in domestic SACs from Bolivia, Peru, and the northwest of Argentina [17,26,30,52,55]. Additionally, macrocysts produced by this parasite were reported in extra-Andean alpacas from the USA and Australia [45,46,60]. Guanaco and vicuña from Argentina were also shown to act as intermediate hosts of *S. aucheniae*, with the formation of macroscopic sarcocysts found in their skeletal muscles [17,29,61].

Microscopic sarcocysts, referred to as *Sarcocystis* sp., *S. lamacanis* or, more recently, *S. masoni*, were reported in the cardiac muscle of llamas and guanacos from Argentina, alpacas from Peru, and alpacas bred in the extra-Andean countries China, Iran, and Australia [17,33,46,62,63,64,65,66]. Microcysts were, in some cases, also observed in other body locations in addition to the myocardium, such as liver, kidney, spleen, lung, tongue, and skeletal muscles [17,33,62,63].

Of the two reported *Sarcocystis* spp. that infect OWC, there is a single report on the identification of *S. ippeni* microscopic sarcocysts in skeletal muscles of the esophagus of two dromedaries of Egypt based on microscopy data [3,19]. On the other hand, several reports describe the presence of microscopic and macroscopic sarcocysts of *S. cameli* or its synonym *S. camelicanis* in dromedaries from Iran, Iraq, Saudi Arabia, and Egypt. The organs and tissues where cysts were found include the esophagus, diaphragm, tongue, heart, and skeletal muscles [19,20,27,28,38,51]. Microcysts identified as *Sarcocystis* spp. were also reported in the same tissues of dromedaries from Egypt, Jordan, Saudi Arabia, Iran, Iraq, Mongolia, and Ethiopia [48,50,53,67,68,69,70,71,72,73]. Unfortunately, nucleotide sequences of taxonomic relevance for *S. ippeni* are missing, as well as species identification in the latter reports. Furthermore, there is scarce information on *Sarcocystis* spp. infections in Bactrian camels and no reports available for *C. ferus* [16,19]. Thus, knowledge on the actual distribution of *Sarcocystis* spp. that infect OWCs is far from complete.

Available studies on the prevalence of camelid sarcocystosis show high-to-very-high values both in SACs and OWCs [38,50,51,52,53,56,57,66,68]. Representative examples of these reports are presented in Table S1.

Regarding risk factors, herd management was considered to influence *Sarcocystis* spp. infection in SACs. In fact, llamas raised in a fenced field with sanitary controls and in the absence of pastoral dogs showed a significantly lower percentage of seropositivity to *Sarcocystis* spp. than those from the same region raised informally by itinerant shepherds without sanitary controls and in the presence of shepherd dogs [57].

In addition, age was found to be a risk factor for sarcocystosis caused by *S. aucheniae* in llamas, *S. masoni* in alpacas, and *Sarcocystis* sp. in dromedaries. In all cases, older age was associated with higher abundances of sarcocysts, most likely due to the prolonged exposure of older animals to infective-stage parasites from the environment [50,52,63,74].

Importantly shepherd dogs, in the case of domestic camelids, and feral or free-ranging dogs, in the case of wild species, that prey on or scavenge on camelids or are fed raw sarcocyst-infected meat can spread infective-stage parasites into the environment [50,68,75].

## 7. Parasite Biology and Host-Pathogen Interaction

As members of the Apicomplexa phylum, *Sarcocystis* protozoa spend most of their life cycle as obligate intracellular parasites. They have evolved sophisticated strategies to invade, live within, and egress from host cells, all of which are essential for their survival and propagation [1]. The elucidation of the molecules and mechanisms involved in the interaction of different-stage pathogens with their hosts could open the way to the rational design of control tools, such as vaccines and chemotherapeutics. For *Sarcocystis* spp., this aspect of research lags behind compared to other pathogenic protozoa due to the scarcity of molecular studies, with only the genome of *S. neurona* sequenced for this genus so far [76].

In the case of *Sarcocystis* spp. infecting camelids, this knowledge gap has been partially filled with the sequencing and partial analysis of the *S. aucheniae* sarcocyst transcriptome, which has allowed for the identification of molecules relevant to the parasite [58,59]. In the first place, in silico analysis of the transcriptome database allowed researchers to identify the biosynthetic pathway of glycosylphosphatidylinositol (GPI), a glycolipid essential for host cell invasion. Glycosylphosphatidylinositol is highly abundant in the membranes of pathogenic protozoa, where it is present as an independent surface molecule or serves as an anchor to surface proteins [77]. Blockade of GPI biosynthesis or treatment of cells with phosphatidylinositol-specific phospholipase C inhibits the in vitro growth of some intracellular pathogenic protozoa, highlighting the vital role of GPIs for these microorganisms [77,78,79]. Moreover, GPIs exert strong immunomodulatory effects during the infection of *Plasmodium falciparum*, which are detrimental to the host [80].

As mentioned above, searches in the *S. aucheniae* transcriptome also identified 24 GPI-anchored proteins that, in addition to their potential usefulness as diagnostic candidates, could also be used for vaccine development [58]. Indeed, vaccine formulations based on the GPI-anchored proteins of various pathogens such as *Trypanosoma cruzi*, *Babesia canis*, and *Schistosoma mansoni* have elicited significant protection upon exposure [81,82,83].

In addition, a number of proteins expressed in *S. aucheniae* sarcocysts were identified by mass spectrometry and transcriptome searches. This approach provided insight into some of the processes that occur within the sarcocyst. Some of the identified proteins predicted to be involved in chromosome separation during mitosis, protein synthesis, and folding can be assigned to the actively dividing metrocyte stage. Other proteins, associated with the specialized secretory organelles of apicomplexans (rhoptries, micronemes, and dense granules) or involved in the process of gliding motility, are expected to be present in the infective stage of the bradyzoite [59]. The aforementioned studies also identified several proteases that can be hypothesized to play essential roles in *S. aucheniae* biology based on what is known about their homologs in other microorganisms [59,84]. Importantly, many of these newly identified parasite proteins represent attractive chemotherapeutic and/or vaccine candidates [59].

Several enzymes involved in respiration (glycolysis, the tricarboxylic acid cycle, and the respiratory chain) and superoxide dismutase, which protects cells from oxidative damage, have been identified among *S. aucheniae* sarcocyst proteins. Thus, it can be concluded that this parasite undergoes aerobic respiration within the sarcocyst, which requires oxygen diffusion from host capillaries through a porous cyst wall [59].

## 8. Phylogeny of Camelids and Camelid-Infecting *Sarcocystis* spp.

**Figure 3:**
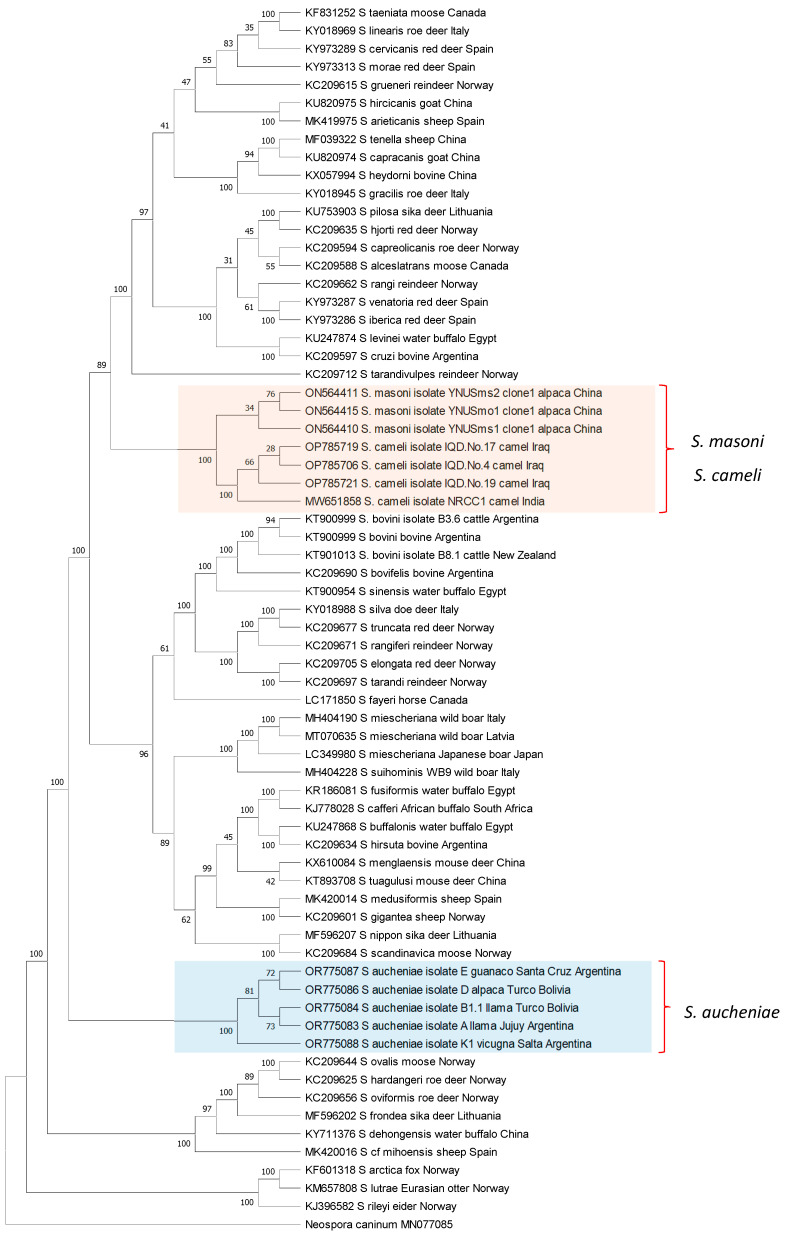
Phylogenetic tree of *Sarcocystis* spp. *cox-1* sequences. The evolutionary history was inferred by using the maximum likelihood method, applying the Kimura 2-parameter model [89]. The analysis involved 68 nucleotide sequences and comprised a total of 946 positions in the final dataset. Bootstrap values are shown close to the branches. *Neospora caninum cox-1* gene sequence was used as the outgroup. The analysis was carried out using MEGA11 [90].

The phylogenetic relationships between *S. aucheniae*, *S. masoni*, and *S. cameli* and with respect to other *Sarcocystis* spp. were investigated by the maximum likelihood (ML) method using available *cox-1* gene sequences (Figure 3). Camelid *Sarcocystis* sequences were segregated into two independent and strongly supported clades: one containing exclusively *S. aucheniae* and the other consisting of two subclades, one of *S. masoni* and another of *S. cameli* sequences. In the inferred tree, *S. masoni* and *S. cameli* represent closely related sister taxons that share a most recent common ancestor. Comparable results were obtained when an ML phylogenetic tree was constructed using 18S rRNA gene sequences. In this case, *S. masoni* and *S. cameli* sequences grouped together in a single clade, clearly separated from the clade of *S. aucheniae* (Figure S1). Notably, as mentioned above, *S. masoni* and *S. cameli* present the same cyst structure (“9j” type of cyst wall, Table 1), further corroborating the notion of a close relationship between these species. Interestingly, *S. aucheniae* appears to be evolutionarily more ancient than most other *Sarcocystis* spp. that infect mammals studied so far.

**Figure 4:**
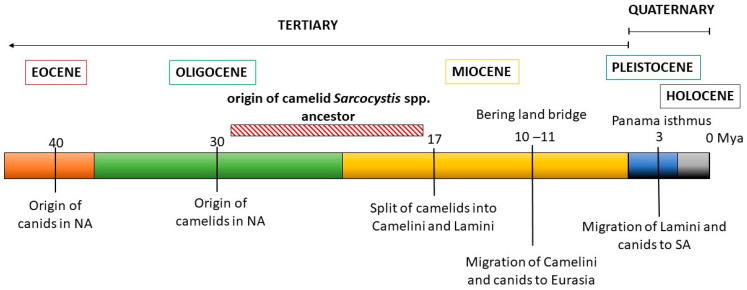
Schematic representation of the evolutionary history of camelids and canids. The likely time period when the ancestor of camelid *Sarcocystis* spp. evolved is shown.

These observations were then contrasted with the evolutionary history of the *Sarcocystis* camelid intermediate and definitive hosts (Figure 4). The ancestral camelid, *Poebrotherium wilsoni*, which resembles a guanaco, originated in North America in the middle Oligocene (25–30 million years ago, mya). Later, in the early Miocene, several changes characteristic to extant camels appeared, such as plantar cushions in each leg, the loss of an upper incisive tooth and the conversion of one of them into a canine, as well as a big depression in the facial part of the maxillary bone to contain the complex lip musculature. During the Miocene, many different groups of camelids evolved, most of which eventually became extinct. One of these groups gave rise to two tribes: Camelini and Lamini, representatives of which migrated through the Bering land bridge to Eurasia and through the Panama land bridge to South America, respectively. The Camelini ancestors diverged to finally give rise to the three *Camelus* spp. that are currently living, around 4.4 mya. Lamini ancestors that arrived in SA belonged to the genus *Hemiauchenia*, which gave rise to the genera *Paleolama*, *Lama*, and *Vicugna*. By the end of the Pleistocene, around 12,000 years ago, *Hemiauchenia* and *Paleolama* had become extinct, while *Lama* and *Vicugna* flourished, particularly in the arid and semiarid regions of the Andes, thanks to their adaptations to thermal stress, dehydration, and hypoxia, as are found in high altitudes [85]. It is accepted that these genera gave rise to two species each: *Lama* to *L. glama* and *L. guanicoe*, and *Vicugna* to *V. pacos* and *V. vicugna*. However, the four species are interfertile and mitochondrial genome data evidence the occurrence of hybridization among them during the process of domestication [86].

Canids (Canidae family) first appeared in North America in the late Eocene (40 mya), evolving from a group of archaic carnivorous mammals, and their evolution is characterized by successive radiations and the occupation of highly diverse niches. In the late Miocene, members of the Caninae subfamily crossed to Eurasia through a land bridge at Bering strait, diversified, and finally gave rise to the modern canids of the Old World, including *Canis familiaris* [87]. In turn, after the Panama isthmus was formed in the Pleistocene, canids arrived in South America, around 3.9 to 3.5 mya, and diversified from a single ancestor, giving rise to numerous species, most of which but four *Lycalopex* spp. became extinct [88].

During the Holocene, around 10 kya, humans accompanied by dogs (*Canis familaris*) crossed the Bering bridge and settled, first in North and then in South America. The Mexican chihuahua and the Peruvian “naked dog” derive from these pre-Columbine lineages. Finally, much later, in the XV century, European colonizers brought with them other *C. familiaris* breeds that spread throughout the Americas [91].

Analyzing these data, two conclusions can be drawn. From the late Oligocene to the early Miocene periods, both ancestral camelids and canids were present in North America before the diversification into and migration of the Camelini and Lamini tribes to Eurasia and South America, respectively (Figure 4). Considering the close relation between *S. masoni* and *S. cameli*, the most recent common ancestor of these *Sarcocystis* spp. must have parasitized a camelid ancestor in the Miocene, using ancient canids as definitive hosts. Thus, the diversification of the ancestral camelid intermediate host into OWCs and SACs resulted in the diversification of an ancestral *Sarcocystis* sp. into *S. cameli* and *S. masoni*, respectively. This coevolutionary pattern is commonly referred to as parasite–host co-speciation. Second, considering that *C. familiaris* became available as a definitive host for SAC *Sarcocystis* spp. only around 5–10 kya, other canids must have previously fulfilled this role after the arrival of camelids to South America 3.9 to 3.5 mya; later, a host shift to *C. familiaris* must have occurred. Extant autochthonous canids whose habitats coincide with those of SACs in the Andean regions include the Culpeo fox (*Lycalopex culpaeus*), the South American gray fox (*Lycalopex griseus*), and, with more limited distribution, the Sechuran fox or Peruvian desert fox (*Lycalopex sechurae*) and Darwin’s fox (*Lycalopex fulvipes*) [88]. A role for these canids as definitive hosts of *S. aucheniae* and/or *S. masoni*, either by predating or—more likely—scavenging on dead SAC carcasses can be hypothesized but awaits experimental confirmation.

## 9. Human Health Implications and Economic Losses Associated with Camelid *Sarcocystosis*

Both OWCs and SACs thrive in desertic areas under harsh conditions, where other types of livestock cannot survive. Their meat constitutes an essential source of animal protein to ensure food safety and poverty alleviation for large human populations living in those environments. Also, the commercialization of camelid meat is an important income source for small family-run producer units which are generally in charge of camelid breeding [9,10,18]. Llama meat is consumed in Bolivia and northwestern Argentina, alpaca meat in Peru, and OWC meat in North Africa, the Middle East, Central Asia, and China [12,13,14].

Camelid meat has a higher protein-to-cholesterol ratio than conventional sources of red meat [12,13,14,92]. This, combined with the lower environmental impact of camelids compared to traditional cattle, meets the profile of environmentally and health-conscious consumers. Thus, SAC meat has attracted the attention of markets outside of South America, as evidenced by the publication of international commercial standards for alpaca and llama meat intended for international markets by the United Nations Economic Commission for Europe (https://unece.org/trade/publications/llamaalpaca-meat-carcases-and-cuts (accessed 13 December 2023)), as well as several publications from Australia dedicated to the production of alpaca meat [93].

Discovery of *Sarcocystis* spp. macroscopic sarcocysts upon slaughter can lead to the condemnation and depreciation of camelid carcasses, significantly hampering the possibilities of formal meat commercialization. In the case of SACs raised in South America, macroscopic sarcocysts due to *S. aucheniae* are a highly common finding in abattoirs [52]. To illustrate the extent of losses caused by SAC sarcocystosis, Peruvian alpaca producers lost around USD 300,000 due to the condemnation and depreciation of carcasses infected with *S. aucheniae* sarcocysts in 1973 [25]. Unfortunately, there are no other available reports on this subject for SACs or OWCs.

To avoid the potential condemnation of SAC carcasses if macroscopic sarcocysts are found, SAC producers in South America frequently avoid abattoirs and resort to slaughtering their animals in their own backyards. After separating part of the meat for the needs of the family, the rest is sold to local butchers, or touristic hotels and restaurants [18]. Preparation of SAC charqui, a traditional salted dried meat product of ancestral origin, is a common practice in the Andean region that allows for the long-term storage of meat at room temperature [92]. Importantly, the informal slaughter of SACs, as well as the processing, storage, and transportation of carcasses, often take place without proper hygienic and sanitary conditions. Thus, although SAC sarcocystosis is not a zoonotic disease, its occurrence results in the informal marketing of SAC meat with its consequent negative implications on food safety [18]. Moreover, it is a common practice to feed house dogs with carcass remains, which can lead to the dissemination of *Sarcocystis* spp. infective forms in the environment through dog feces and the perpetuation of the parasite cycle [1].

Recently, the commercialization of guanaco meat was approved in the southern regions of Argentina and Chile. However, the high numbers of animals presenting *S. aucheniae* macroscopic sarcocysts upon slaughter constitute a main constraint for this market of regional and international interest [94,95,96]. The reported positivity rates of sarcocysts in guanaco tissues in Argentina and Chile varied between 69 and 100% [94,95].

It has been proposed that human’s consumption of raw or insufficiently cooked *Sarcocystis*-infected camelid meat leads to gastroenteritis signs due to the ingestion of sarcocystin toxins, although there are no published human case reports [25]. Importantly, when infected meat was cooked by boiling, grilling, or frying, the toxin was inactivated, as assessed in experiments with rabbits inoculated with sarcocyst homogenates. The preparation of charqui, on the other hand, prevented parasite transmission to dogs but did not eliminate sarcocyst toxic effects on rabbits [40,41].

Finally, there is no available information on the effects of sarcocystosis on the productivity parameters of OWCs and SACs.

## 10. Conclusions and Perspectives

Sarcocystosis is an old and well-known problem for domestic SAC and OWC meat commercialization. Prevalence in herds is high, and tissues are heavily infected with cysts. Although infections are generally subclinical, occasional pathogenicity is observed, while the effects of sarcocystosis on the well-being and fitness of domestic and wild camelids is unknown. Diagnostic methods that can be reliably applied in live animals are not available, and no tools or therapies to control these infections have so far been designed. Currently, dogs appear to be the main definitive hosts for at least three of the *Sarcocystis* spp. that infect camelids. The close relationship between camelid shepherds and dogs, resulting in their frequent feeding with raw camelid meat and the presence of feral and free-roaming dogs in camelid breeding areas, make it difficult to break the parasite life cycle through parasite control in dogs.

*Sarcocystis* spp. have developed sophisticated adaptations to perpetuate themselves in their hosts along millions of years of evolution. Recent research has started to fill the gap in knowledge on the molecules of camelid *Sarcocystis* spp. parasites that are essential for the host–pathogen interactions and that constitute attractive targets for the development of vaccines and therapeutic interventions. These research efforts, as well as increased knowledge on the epidemiology of these parasites and novel diagnostic tests, will undoubtedly result in a breakthrough for camelid-breeding communities.

## Figures and Tables

**Table 1:** Characteristics of sarcocysts produced by camelid-infecting *Sarcocystis* sp.

Intermediate Host	Species	Sarcocyst					Ref.
		Shape, Size (Length × Width)	Cyst Wall			Location	
			Type	Thickness (µm)	Villar Protrusions (vp) Size and Aspect		
OWC	*S. cameli*	Fusiform, microscopic (700 × 100 µm) and macroscopic (1.5–5.0 × 0.2–0.4 mm)	9j	<2	3.0 × 0.5 µm finger-like vp	cardiac and skeletal muscle	[3,19,20,27,28]
	*S. ippeni*	Globular, microscopic (100–120 × 50–100 µm)	32	2.3–3.0	1.0 × 0.25–1.2 µm thorn-like vp	skeletal muscle	[3,19]
SAC	*S. aucheniae*	Oval, macroscopic (0.5–2.0 × 0.2 cm)	21	6–10	3–4.5 × 2.5–3.5 µm branched vp, cauliflower-like wall	skeletal muscle	[1,17,26,29,30]
	*S. masoni*	Fusiform, microscopic (800 × 95 µm)	9j	2.5–3.5	2–2.8 × 0.5–0.7 µm conical to cylindrical vp	cardiac and skeletal muscle	17

## Data Availability

Publicly available datasets were analyzed in this study. Sequences included in the phylogenetic trees can be found at: https://www.ncbi.nlm.nih.gov/genbank/ (accessed on 13 December 2023), using the accession numbers included in the trees.

## Supplementary Materials

The following are available online at https://www.mdpi.com/article/10.3390/pathogens13030196/s1, Table S1: Epidemiological studies of *Sarcocystis* spp. infecting South American (SAC) and Old-World camelids (OWC), Figure S1: Phylogenetic tree of *Sarcocystis* spp. 18S rRNA sequences.
