# Supplementary material for: Sarcocystis spp. of New and Old World Camelids: Ancient Origin, Present Challenges
Source: Pathogens. 2024 Feb 23;13(3):196. doi: 10.3390/pathogens13030196 (PMC10975914; doi:10.3390/pathogens13030196)

Table S1. Epidemiological studies of *Sarcocystis* spp. infecting South American (SAC) and Old-World camelids (OWC).

**Host**	***Sarcocystis* sp.**[1]	**Intermediate host**	**Examined tissue / organ**	**Diagnostic method**[2]	**n′**	**Positivity (%)**	**Geographic location**	**Ref.**
SAC	*S. aucheniae*	llama	skeletal muscles of entire carcasses	GI	1196	34	Southern High Plateau, Bolivia	52
			intercostal and cervical muscles	GI	80	65	Jujuy, Argentina	55
		guanaco	tongue, heart, skeletal muscles, fascias	GI, HIS	12	66	Chubut, Argentina	62
	S. masoni	alpaca	heart	HIS	104	100	Huancavelica, Peru	66
					60	100	Puno, Peru	65
	anti-*Sarcocystis sp.* antibodies	llama	serum	IFAT	308	96	Jujuy, Argentina	56
				iELISA	507	36	Jujuy, Entre Rios and Buenos Aires, Argentina	57
OWC	*S. cameli*	one-humped camel	esophagus, heart, diaphragm, tongue, skeletal muscles	SQ	37	40	Riyadh and Dammam, Saudi Arabia	27
			esophagus, heart, diaphragm, tongue	HIS	180	64	Cairo, Beni, Suef and El Minia, Egypt	38
			esophagus, heart, diaphragm, tongue, skeletal muscles	SQ	50	42	Riyadh and Al Qassim, Saudi Arabia	51

			esophagus, heart, diaphragm	ED	103	80	Al-Ahsa, Saudi Arabia	48
			esophagus, heart, diaphragm	ED	36	92	Baghdad, Iraq	67
			esophagus, heart, diaphragm, masseter muscle, shoulder	HIS	121	45	Borana and Liben, Ethiopia	68
	Sarcocystis sp. microscopic cysts		esophagus, heart, diaphragm, tongue, rectus femoris muscle	SQ	400	52	Najaf-Abad, Iran	70
			esophagus, heart, diaphragm, tongue, masseter muscle	HIS	250	84	Khorasan Province, Iran	50
			esophagus, heart, and masseter, intercostal and limb muscles	ED	130	51	Yazd Province, Iran	71
			esophagus, heart, diaphragm	SQ/ED	100	75	Daraw Aswan Governorate, Egypt	53

[1]*Sarcocystis* spp. names are according to the currently accepted taxonomy, independently of the name used in the publication (*S. lamacanis* = *S. masoni*; *S. camelicanis* = *S. cameli*)
[2]Diagnostic procedure employed to obtain the reported positivity percentages. GI: detection of macroscopic sarcocysts by gross inspection; HIS: detection of microscopic sarcocysts by observation under light microscopy of stained thin sections of paraffin-embedded tissues; SQ: squashing of tissues with a glass slide followed by observation of microscopic sarcocysts by light microscopy; ED: enzymatic digestion of tissues followed by observation of microscopic sarcocysts and released parasites by light microscopy.

Figure S1. Phylogenetic tree of *Sarcocystis* spp. 18S rRNA sequences. The evolutionary history was inferred by using the maximum likelihood applying the Kimura 2-parameter model [88]. The analysis involved 85 nucleotide sequences and comprised a total of 531 positions in the final dataset. Bootstrap values are shown close to the branches. *Toxoplasma goondii* 18S rRNA gene sequence was used as outgroup. The analysis was carried out using MEGA11 [89].

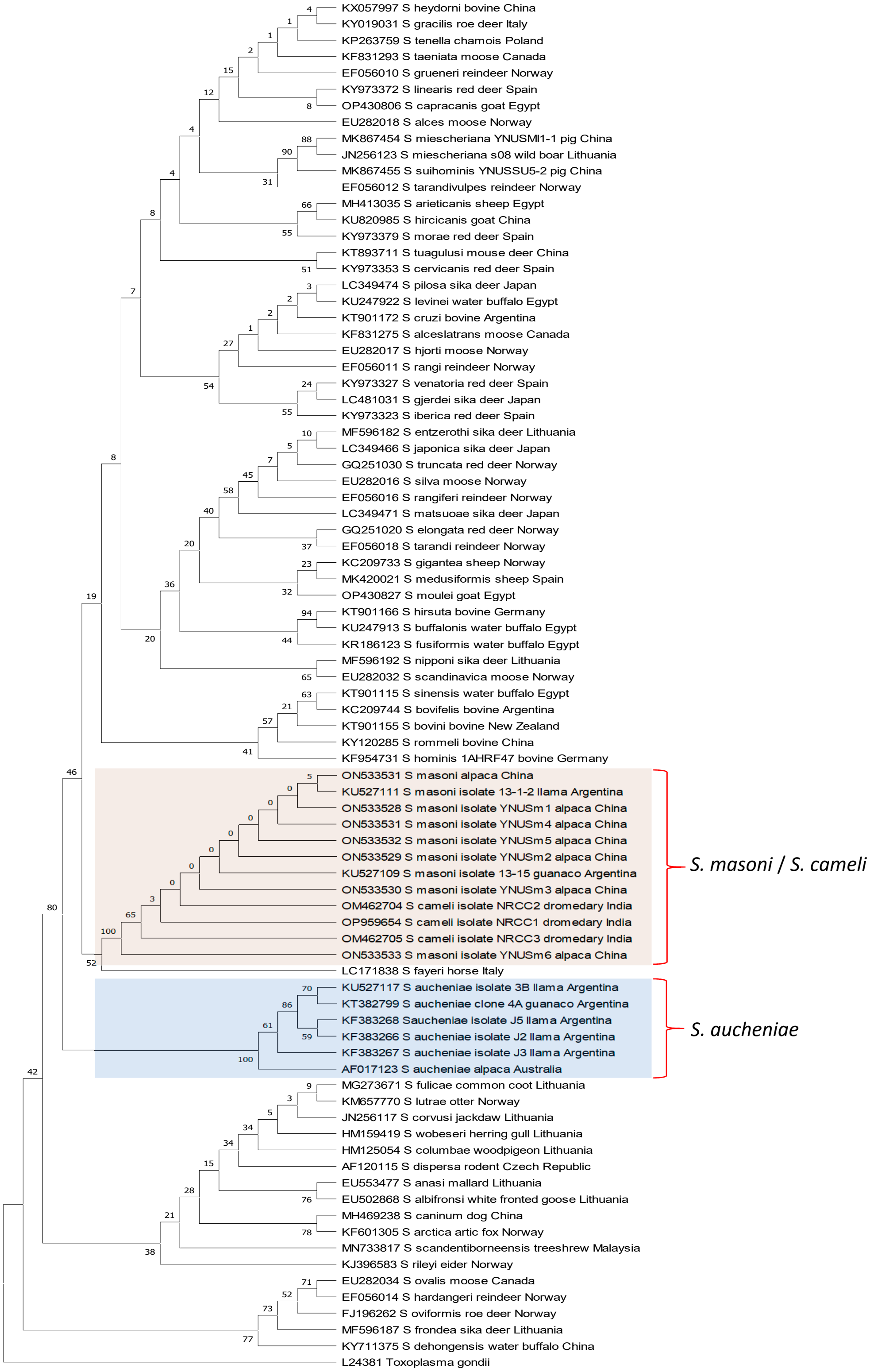

Supplement: Supplementary file 1 [file pathogens-13-00196-s001.zip › pathogens-2828266-supplementary.pdf]
